# Postbiotic Metabolite Derived from *Lactiplantibacillus plantarum* PD18 Maintains the Integrity of Cell Barriers and Affects Biomarkers Associated with Periodontal Disease

**DOI:** 10.3390/antibiotics13111054

**Published:** 2024-11-06

**Authors:** Widawal Butrungrod, Chaiyavat Chaiyasut, Netnapa Makhamrueang, Sartjin Peerajan, Wantida Chaiyana, Sasithorn Sirilun

**Affiliations:** 1 Department of Pharmaceutical Sciences, Faculty of Pharmacy, Chiang Mai University, Chiang Mai 50200, Thailand; widawal_b@cmu.ac.th (W.B.); chaiyavat@gmail.com (C.C.); netnapa.ma@cmu.ac.th (N.M.); wantida.chaiyana@cmu.ac.th (W.C.); 2 Innovation Center for Holistic Health, Nutraceuticals, and Cosmeceuticals, Faculty of Pharmacy, Chiang Mai University, Chiang Mai 50200, Thailand; 3 Office of Research Administration, Chiang Mai University, Chiang Mai 50200, Thailand; 4 Health Innovation Institute, Chiang Mai 50200, Thailand; s.peerajan@gmail.com

**Keywords:** *Lactiplantibacillus plantarum*, postbiotics, postbiotic metabolite, cell barrier integrity, tight junction, periodontal disease, inflammation

## Abstract

**Background/Objectives**: Periodontal disease is caused by oral infections, biofilms, persistent inflammation, and degeneration of cell barrier integrity, allowing pathogens to invade host cells. Probiotics have been extensively studied for the treatment of periodontal disease. However, research on the involvement of beneficial substances produced by probiotics, called “postbiotics,” in periodontal diseases remains in its early stages. The present study aimed to evaluate the effect of a postbiotic metabolite (PM) from *Lactiplantibacillus plantarum* PD18 on immunomodulation and maintenance of cell barrier integrity related to periodontal disease. **Method**: The main substance in PM PD18 was analyzed by GC-MS. The cytotoxic effect of PM PD18 was performed using the MTT assay, wound healing through the scratch assay, cell permeability through TEER value, modulation of inflammatory cytokines through ELISA, and gene expression of inflammatory cytokines and tight junction protein was determined using qRT-PCR. **Results**: The main substance found in PM PD18 is 2,3,5,6-tetramethylpyrazine. PM PD18 did not exhibit cytotoxic effects on RAW 264.7 cells but promoted wound healing and had an antiadhesion effect on *Porphyromonas gingivalis* concerning SF-TY cells. This postbiotic could maintain cell barrier integrity by balancing transepithelial electrical resistance (TEER) and alkaline phosphatase (ALP) activity. In addition, the gene and protein expression levels of zonula occludens-1 (ZO-1) increased. PM PD18 was found to have immunomodulatory properties, as demonstrated by regulated anti- and pro-inflammatory cytokines. Interleukin-10 (IL-10) increased, while IL-6 and IL-8 were reduced. **Conclusions**: This study demonstrated that PM PD18 is efficient as a natural treatment for maintaining cell barrier integrity and balancing inflammatory responses associated with periodontal disease.

## 1. Introduction

Periodontal diseases are degenerative processes caused by infections and inflammation affecting the periodontium, which is the tissue that surrounds and supports the teeth [1,2]. If left untreated, periodontitis contributes to advancing damage to the tooth-attachment apparatus known as the periodontium (gingiva, cementum, periodontal ligament, and alveolar bone), compromises mastication and aesthetics, and negatively impacts quality of life [3,4,5]. Periodontitis is the sixth most common illness globally, affecting around 10% of adults [6]. The most frequent types of periodontal disease are gingivitis and periodontitis. These disorders are caused by oral bacteria that create biofilms on the surfaces of teeth and in the periodontal pocket, leading to inflammatory responses [2,7]. Dysbiotic bacterial populations in the mouth can encourage an expansion of bacteria related to periodontal disease, including *Porphyromonas gingivalis*, *Aggregatibacter actinomycetemcomitans*, *Treponema denticola*, *Prevotella loescheii*, *Prevotella intermedia*, *Fusobacterium nucleatum*, and *Tannerella forsythia* [1]. In this setting, *P. gingivalis*, a black-pigmented Gram-negative bacteria, is classified as a late colonizer. It is frequently found in coaggregation with primary and secondary colonizers in the top layer. Whereas most oral microorganisms are considered commensal, others, such as *P. gingivalis*, are regarded as opportunistic or keystone pathogens [8]. To colonize the periodontium and spread to the subgingival area, *P. gingivalis* must overcome the host’s immunological systems. This pathogen could affect the host’s response to inflammation to evade immune responses and prevent bacterial death. Deficient immune responses promote a pro-inflammatory environment favoring constant infection, which is characteristic of chronic periodontitis [8]. Infections in the oral cavity promote production of several pro-inflammatory cytokines, including interleukin-6 (IL-6), IL-1ß, IL-8, and tumor necrosis factor alpha (TNF-α). If these cytokines are produced in excess or imbalance, it may lead to injury and ultimately bone resorption and tooth loss [9,10,11,12]. Lipopolysaccharide (LPS), a specific element of the cell walls of Gram-negative bacteria and a main initiator of inflammation, increases the fast production of pro-inflammatory cytokines or inflammatory mediators. Consequently, LPS has been routinely used to develop experimental models of periodontitis [13,14].

Barrier integrity plays an important role in avoiding microbial invasion. It is mostly dependent on tight junctions consisting of proteins such as zonula occludens-1 (ZO)-1, occludin, claudins, adherens junctions, and gap junctions [15]. These complexes form homotypic cell–cell attachments, collectively providing a physical barrier against pathogens [16]. *P. gingivalis* has been reported to degrade these intercellular junctional complexes, thereby compromising this critical cell barrier function and allowing *P. gingivalis* to reach the underlying connective tissue. This promotes bacterial colonization of the periodontium, a significant phase in the progression of periodontitis [8].

Mechanical plaque removal is a conventional therapy for these disorders, along with antibiotics and chemical agents like chlorhexidine [1]. However, these treatments have side effects such as pain, edema, tooth sensitivity, discoloration, and medication resistance [17]. Accordingly, contemporary therapies incorporating natural advantageous microbial categories, including probiotics, have advantages desired for regulating the oral microbial community, decreasing infections in the mouth, and stimulating the host’s immune system reactions [18,19,20]. Nevertheless, their application as products remains limited. Probiotics’ live cells can trigger fermentation processes, changing the chemical and physical properties and longevity of the items. Probiotics can become inactive and may have harmful consequences in immunocompromised people [1,21,22,23,24,25].

To circumvent these constraints, probiotics create helpful compounds known as “postbiotics”, which can be employed as an alternate strategy. “Postbiotic” describes the creation of inanimate microbes or their components, which provide health benefits to humans [26]. They have garnered interest due to their great stability, safe administration settings, and longer storage periods, giving them an extensive range of promise in the sectors of food and medicine [21]. Many studies have indicated that postbiotic metabolites (PMs) from beneficial bacteria, such as probiotics and lactic acid bacteria (LAB), may provide protection against pathogens. One related study revealed that metabolites produced by *Lactobacillus* spp. exhibit antagonistic properties against the biofilm formation of several pathogenic bacteria. Furthermore, they have been demonstrated to be useful for reducing tooth decay. These findings are congruent with another study, which demonstrated that Probio-MT had a significant suppressive impact on oral pathogenic microbes, including *Streptococcus mutans*, *A. actinomycetemcomitans*, *P. gingivalis*, and *F. nucleatum*, and may serve as an additional therapy for dental caries and periodontal disease, along with other oral diseases [21,27].

In agreement with related research, our earlier study revealed that PMs from *Lactiplantibacillus plantarum* (formerly *Lactobacillus plantarum*) PD18 have antimicrobial and antibiofilm activities against bacteria-related periodontal diseases, including *P. gingivalis* ATCC 33277, *T. forsythia* ATCC 700191, *P. loescheii* ATCC 15930, and the biofilm-associated oral pathogen *S. mutans* ATCC 25175. The purpose of this study was to examine the influence of a postbiotic metabolite from *L. plantarum* PD18 (PM PD18) concerning safety evaluations for cells, wound healing, the adhesion of *P. gingivalis* to cells, barrier integrity improvement, and immunomodulation properties.

## 2. Results

### 2.1. Identification of PM PD18 Using GC–MS and HPLC

**Figure 1:**
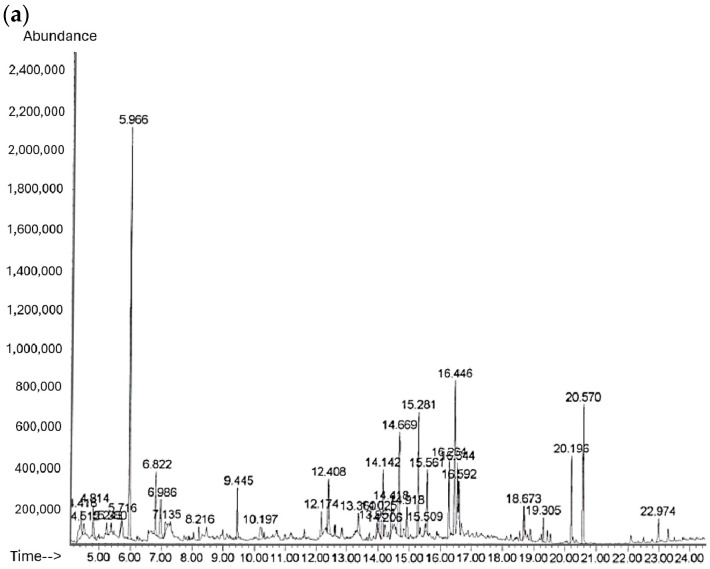

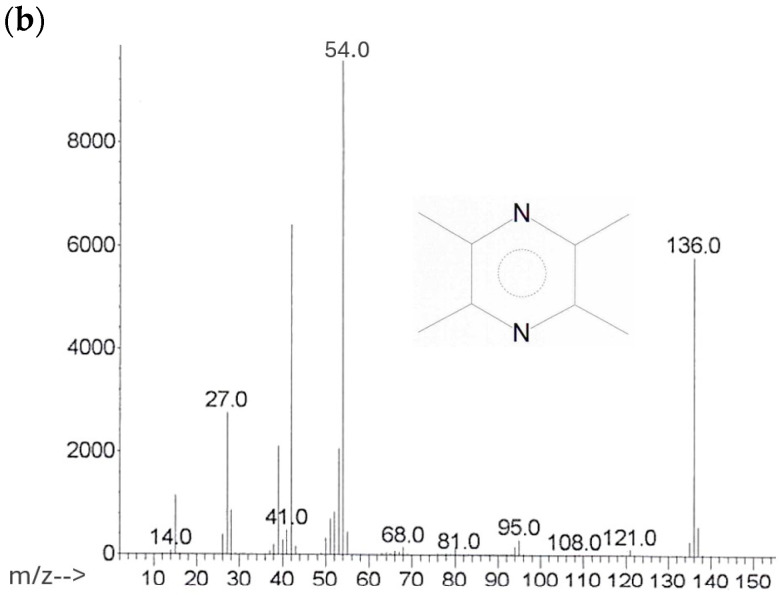
(**a**) GC-MS chromatograms of compounds in PM PD18. (**b**) Mass spectrum of peak at retention time of 5.966 min in PM PD18 as analyzed using NIST.

The compounds in PM PD18 were determined using GC-MS. A chromatogram of numerous components in PM PD18 is shown in Figure 1a. The chromatogram shows the major peak at the retention time of 5.966 min that was used in further analysis. The mass spectrum of the major peak was compared with the NIST standard library to confirm its identity. It was obviously similar to 2,3,5,6-tetramethylpyrazine (TMP), with the same base peak at *m*/*z* 54 and other major peaks at *m*/*z* 136 (Figure 1a). The mass spectrum and the structure are shown in Figure 1b.

An HPLC analysis of the major compound of PM PD18 is shown in Table 1. We did not find TMP at 0 h, but we did find it after 72 h of incubation, with a retention time of 8.896 min and a concentration of 0.1% (*w*/*w*). This result indicates that TMP was not a pre-existing or present substance in the culture medium and may have been derived from the fermentation process of *L. plantarum* PD18. In addition, the amount of TMP found in PM PD18 was 0.1 g of TMP/g of freeze-dried PM PD18 powder.

### 2.2. Safety Assessment of PM PD18

#### 2.2.1. Effect of PM PD18 on Viability of RAW 264.7 Cells

**Figure 2:**
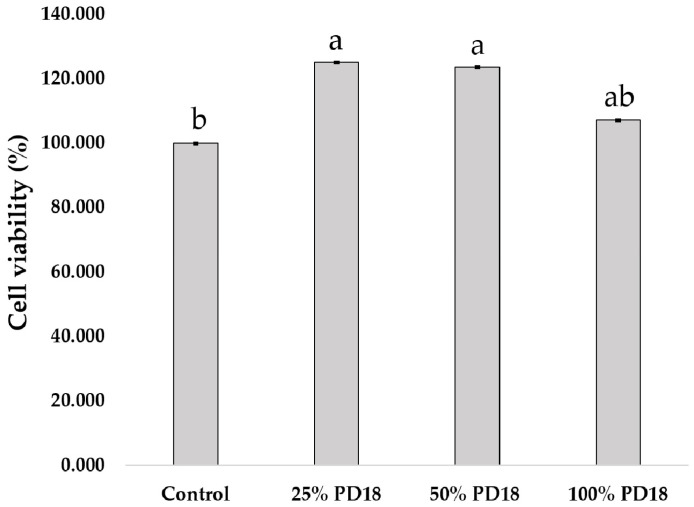
The effect of PM PD18 on the viability of RAW 264.7 cells. The means ± standard deviations of three experiments are displayed. Different letters above the bars indicate significantly different (*p* < 0.05) values.

The effect of PM PD18 on the viability of RAW 264.7 cells was our first area of investigation regarding safety. The percentage of cell viability after treatment with PM PD18 is shown in Figure 2. The highest percentages of cell viability were found in 25 and 50% PM PD18 (125.08 ± 8.14% and 123.50 ± 11.74%, respectively), with no significant difference between these two groups. The 100% PM PD18 had 107.22 ± 1.63% cell viability with no significant difference compared with the control. The results revealed that the RAW 264.7 cells were not cytotoxically affected by PM PD18.

#### 2.2.2. Effect of PM PD18 on Wound Healing

**Figure 3:**
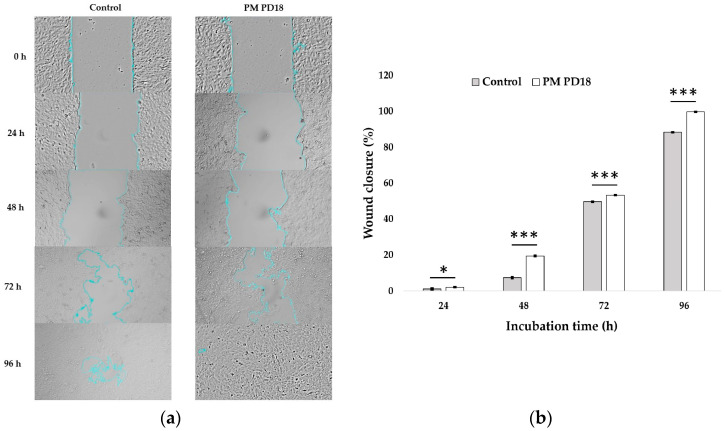
The migration of SF-TY cells with or without PM PD18. (**a**) Images of the migration of SF-TY cells were observed at 0, 24, 48, 72, and 96 h. (**b**) The wound closure percentages of the SF-TY cells treated with PM PD18 were compared with those of the control at different incubation times. These data represent the means ± SDs of three experiments. * and *** indicate *p* < 0.05 and *p* < 0.001, respectively, compared with the control.

The purpose of the scratch experiment was to study cell migration, which is a key part of wound healing. The migration of SF-TY cells with or without PM PD18 is shown in Figure 3a. The wound closure percentages of untreated SF-TY cells and cells treated with PM PD18 steadily increased as the incubation period was extended, as shown in Figure 3b. The wound closure percentages in the PM PD18-treated cells were significantly higher than those of the control at all time points. According to these findings, PM PD18 promoted wound healing in a time-dependent manner and had a positive effect compared with the control.

### 2.3. Effect of PM PD18 on Adhesion of Pathogens to SF-TY Cells

In this investigation, the capacity of PM PD18 to inhibit the adherence of the periodontal pathogen strain *P. gingivalis* to cells was assessed. The result showed that the percentage of adherence inhibition of *P. gingivalis* adhering to SF-TY cells was significantly higher for 50% PM PD18 than 25% (78.75 and 61.95%, respectively). This result revealed that PM PD18 decreased the adherence of *P. gingivalis* in a dose-dependent manner.

### 2.4. Effect of PM PD18 on Induced SF-TY Cell Monolayer Permeability

**Figure 4:**
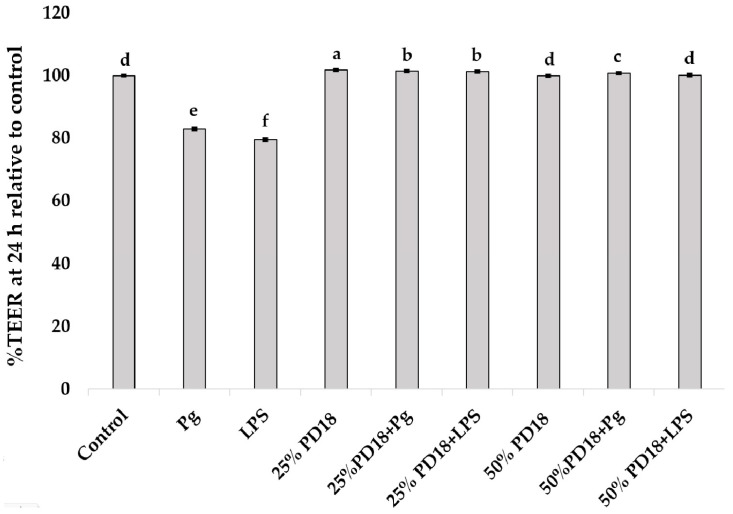
The effect of PM PD18 co-cultured with *P. gingivalis* and LPS on SF-TY cell monolayer permeability. These data represent the means ± SDs of three experiments. Different letters above the bars indicate significantly different (*p* < 0.05) values.

TEER values, serving as markers of permeability variance, were measured before treatment and over the next 24 h. At 24 h, the TEER values of all samples were similar to those of the control except the SF-TY cells treated with LPS and *P. gingivalis* (Figure 4). The cells treated with *P. gingivalis* and LPS showed a significant decrease in %TEER relative to the control (83.01 ± 0.11 and 79.06 ± 0.15, respectively). The cells in the PM PD18 groups showed significantly higher %TEER values relative to control than those in the LPS and *P. gingivalis* groups. This finding revealed that PM PD18 has a positive effect on cell permeability that may maintain or improve cell barrier integrity. 

### 2.5. Effect of PM PD18 on Alkaline Phosphatase (ALP) Activity of SF-TY Cells

**Figure 5:**
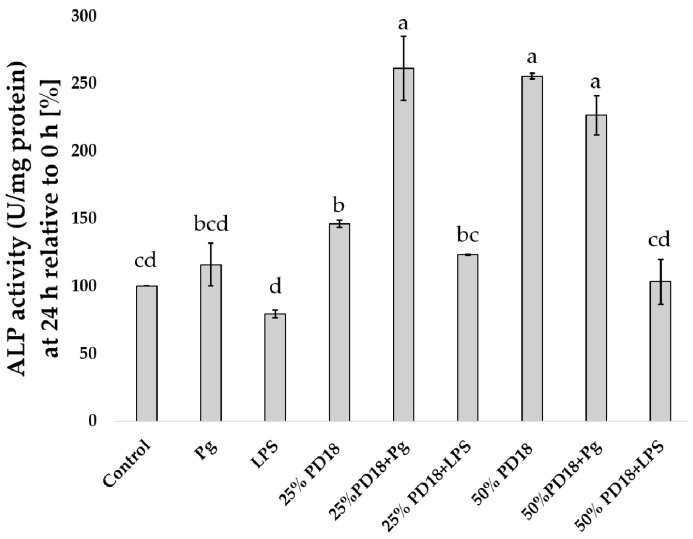
The ALP activity of SF-TY cells co-cultured with PD18, *P. gingivalis,* and LPS. These data represent the means ± SDs of three experiments. Different letters above the bars indicate significantly different (*p* < 0.05) values.

After SF-TY cells were treated with *P. gingivalis*, LPS, and PM PD18, their ALP activity was measured. The results showed that treatments with 25% PM PD18 and *P. gingivalis*, 50% PM PD18, and 50% PM PD18 and *P. gingivalis* had ALP activities at 24 h (261.34 ± 23.82%, 255.51 ± 13.49%, and 226.53 ± 14.44%, respectively, relative to 0 h) that were significantly higher than the other treatments (Figure 5). While the lowest ALP activity was found in LPS-treated cells (79.53 ± 2.83%), no significant difference was noted when compared with the control and 25% PM PD18 with LPS.

### 2.6. Modulation of Inflammatory Cytokines in SF-TY Cells Using PM PD18

To determine the effect of PM PD18 on the cytokine levels (IL-6, IL-8, and IL-10) in SF-TY cells challenged with *P. gingivalis* and LPS, we co-cultured the cells with *P. gingivalis* and LPS with or without 25 or 50% PM PD18 and evaluated secreted cytokines by ELISA. Table 2 shows that 25 and 50% PM PD18 significantly attenuated pro-inflammatory cytokine secretion, including IL-6 and IL-8, compared with *P. gingivalis-* and LPS-treated cells without PM PD18. We found an interesting result that no IL-6 cytokine secretion was detected in cells treated with 50% PM PD18, although IL-6 was measured at 1.17 ± 0.10 in the control. The results also showed that 50% PM PD18 could reduce IL-6 levels significantly compared to 25% PM PD18. In contrast, 25% PM PD18 significantly reduced IL-8 levels compared to 50% PM PD18, *P. gingivalis,* and LPS without PM PD18.

Considering the secretion of the anti-inflammatory cytokine IL-10, 50% PM PD18 showed the highest IL-10 level at 92.18 ± 0.10 pg/mL, which was significantly higher than the levels of the other groups. The 25% PM PD18 group had the second-best result at 15.04 ± 0.09 pg/mL, whereas *P. gingivalis-* and LPS-treated cells did not exhibit IL-10 secretion. These results indicated that both 25 and 50% PM PD18 could modulate the pro-inflammatory and anti-inflammatory cytokine secretion levels of cells co-cultured with *P. gingivalis*, LPS, and PM PD18.

### 2.7. Effect of PM PD18 on Inflammatory Cytokines and Tight Junction Protein Gene Expression

**Figure 6:**
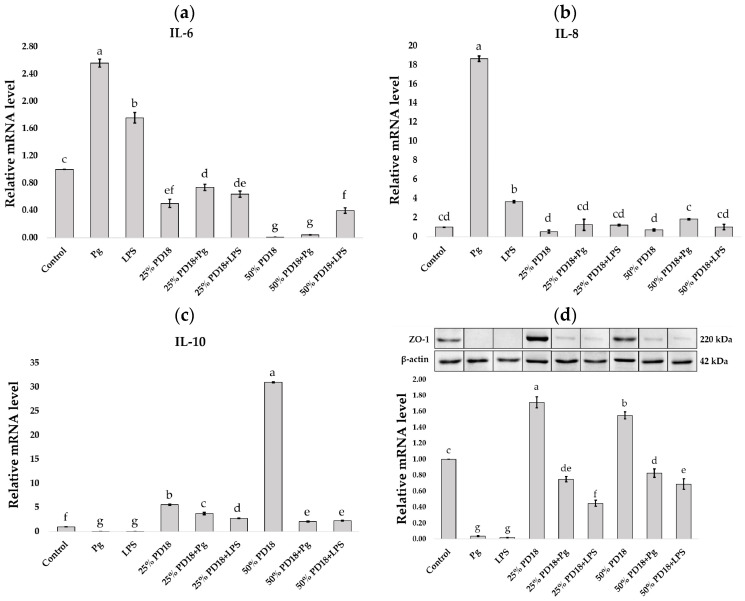
The effects of 25 and 50% PM PD18 on inflammatory cytokines and tight junctions in *P. gingivalis-* and LPS-induced SF-TY cells. The relative gene expression of (**a**) *IL-6*, (**b**) *IL-8*, (**c**) *IL-10*, and (**d**) *ZO-1* was determined and normalized with β-actin. (**d**) Western blotting for ZO-1 and β-actin in SF-TY cells. These data represent the means ± SDs of three experiments. Different letters above the bars indicate significantly different (*p* < 0.05) values.

In this study, the gene expression of the pro-inflammatory cytokines IL-6 and IL-8, the anti-inflammatory cytokine IL-10, and the tight junction protein ZO-1 in SF-TY cells was determined using qRT-PCR after they were co-cultured with *P. gingivalis* and LPS with or without 25 or 50% PM PD18. The results demonstrated that PM PD18 produced a direct effect on the substantial levels of pro-inflammatory and anti-inflammatory cytokines, as displayed in Figure 6a–c. The samples co-cultured with 25 or 50% PM PD18 with or without *P. gingivalis* and LPS showed significant downregulation of IL-6 expression (*p* < 0.05) compared with the control (Figure 6a). For IL-8, a clear trend towards significantly decreased expression was also observed in the PM PD18-treated groups when compared with cells co-cultured with *P. gingivalis* and LPS without PM PD18, which were not significantly different from the control (Figure 6b). However, upregulation of IL-10 gene expression was found in all treatment groups co-cultured with PM PD18 when compared with the control, *P. gingivalis,* and LPS groups without PM PD18, as shown in Figure 6c. For the tight junction protein ZO-1, the 25 and 50% PM PD18-treated cells revealed significant upregulation of ZO-1 gene and protein expression that was significantly increased when compared with the other treatments (Figure 6d). The above results revealed that the levels of pro-inflammatory cytokines, anti-inflammatory cytokines, and tight junction proteins in untreated SF-TY cells and cells treated with *P. gingivalis* and LPS may be modulated after exposure to PM PD18.

## 3. Discussion

Periodontal disease is now commonly recognized as being predominantly caused by bacterial infection and the host’s inflammatory response [2,7,28]. Pathogenic bacterial infections occur when bacteria attach to the teeth in the form of dental biofilms. They interact with one another and the host. Over time, a dysbiotic microbiota, along with imbalanced host inflammation, promotes the development of certain microorganisms inside the biofilms, producing substances that increase inflammation, leading to tissue degradation and tooth loss [7]. In terms of bacterial infection and biofilm formation, our related study found that PM PD18 has antimicrobial and antibiofilm activities against periodontal pathogens, including *P. gingivalis* ATCC 33277, *T. forsythia* ATCC 700191, *P. loescheii* ATCC 15930, and the biofilm-associated oral pathogen *S. mutans* ATCC 25175 [1]. However, the effects of PM PD18 on the safety assessments of cells, pathogen adhesion to cells, wound healing, barrier integrity, and immune responses remained undetermined.

The results of the present study demonstrated that PM PD18 is mainly composed of TMP. TMP is an organic compound from the pyrazine family. It was shown to have several pharmacological properties such as antioxidant, anti-inflammatory, and anti-cerebral ischemia properties [29] and has been widely utilized in China to treat a variety of disorders, including cardiovascular and cerebrovascular diseases [11,14,30,31]. TMP may be generated through microbial metabolism under proper fermentation conditions, resulting in biotransformation, and it is generated by the condensation of acetoin and ammonia, which serve as precursors to TMP [31,32]. Acetoin is created by fermenting glucose, the main source of carbon and a component of the MRS medium, into pyruvate, which may subsequently be transformed into acetoin via the diacetyl–acetoin pathway. Whereas ammonium (or ammonia) is produced by the decomposition of arginine in peptones, the yeast extract in the MRS medium contributes nitrogen and a basic structure to TMP. Chemical interactions between carbonyl compounds (acetoin) and amino acids culminate in the formation of the pyrazine structure [31,33,34].

Assessing the safety of active ingredients is critical before they are used in oral or personal care products. Postbiotics target a wide range of areas, including the oral cavity, skin, genitourinary tract, and nasopharynx. As a result, postbiotics may be used in several sectors, including food and medicine. Postbiotics from probiotics are generally considered safe due to their clear genetic backgrounds and biological properties [21]. The findings of the present study revealed that PM PD18 had no cytotoxic effects on SF-TY cells and possessed wound healing properties. Surgical removal of lesions, recurring ulcers, and radiation injuries are major causes of oral wounds, often resulting in oral mucosa and soft tissue deficiencies [13]. Therefore, several techniques for supporting periodontal tissue regeneration, as well as medications that improve wound healing, have been investigated [35]. PM PD18 enhanced wound healing in a time-dependent manner and had a beneficial impact compared to the control.

In periodontal disease, *P. gingivalis* interrupts the homeostatic balance by adhering to earlier colonizers, shifting the commensal microbial community to a pathogenic state. It plays a role in the pathogenesis and progression of periodontitis and has been identified as a keystone pathogen [8,13,36]. Therefore, in this investigation, we focused on *P. gingivalis* as the main pathogen and studied the effect of PM PD18 on the adhesion of *P. gingivalis* to SF-TY cells. Bacterial adhesion, a crucial stage of bacterial infection, allows bacteria to remain in the extracellular environment, colonize, and eventually internalize to evade host defenses, and antiadhesion treatment is effective in preventing or treating bacterial infections [37,38]. In the present study, we found that PM PD18 decreased the adhesion of *P. gingivalis* in a dose-dependent manner. One of the mechanisms that has been proposed for the antiadhesive capacity of postbiotics is that postbiotics prevent harmful microbes from colonizing carrier surfaces by altering their adherence attributes. One related study reported that *Lactobacillus parasitum* HL32’s metabolite was shown to destroy *P. gingivalis*, whereas heat-inactivated *Lactobacillus* sp. exhibited antiadhesion and antibiofilm characteristics against cariogenic oral bacteria. This had a good influence on the oral microflora without extra risks compared with live bacteria [21]. The surface adhesin of *Lacticaseibacillus rhamnosus* could compete with oral streptococci, such as *S. mutans*, for saliva receptors, limiting the adherence and biofilm development of dangerous bacteria [39].

In addition to *P. gingivalis*, we also used LPS, a component of Gram-negative bacteria’s cell walls that may cause immunologic activation and adverse pathophysiologic consequences in the body [13,14], as a crucial pathogenic element in the study of barrier integrity and the immune response. The importance of cell barrier integrity in the oral cavity lies in its role in preventing foreign substances such as bacteria, viruses, and potentially harmful chemicals from entering the body through the oral mucosa. Maintaining the integrity of the cell barrier ensures that the oral mucosa functions as an effective protective barrier, helping prevent infections and inflammation and reducing the risk of oral diseases such as periodontal disease and dental caries. Additionally, cell barrier integrity plays a crucial role in maintaining the microbial balance in the oral cavity and aids in the processes of wound repair and healing within the mouth [40,41]. A common and simple measurement of epithelial permeability and integrity is offered by TEER. This study found that *P. gingivalis* and LPS significantly reduced TEER. However, co-culturing with PM PD18 and PM PD18 alone had no effect on the SF-TY cells’ TEER values. This result corresponds with a related study by Liu et al. [42], which indicated that *L. reuteri* could maintain the TEER values of IPEC-J2 cells by retaining the TEER values in a group of enterotoxigenic *Escherichia coli* (ETEC) K88 incubated with the *L. reuteri*.

To further understand how PM PD18 protects the cell barrier function, we examined the tight junction protein ZO-1 and its transcripts in SF-TY cells. Tight junction proteins are essential for maintaining the membrane barrier’s function and integrity [43]. After treating with *P. gingivalis* and LPS, we found significant reductions in ZO-1 expression and transcripts in SF-TY cells. In contrast, co-culturing with PM PD18 significantly reduced these decreases. The results of this experiment are consistent with related research showing that *L. plantarum* may sustain tight junction protein expression, possibly protecting the intestinal barrier from ETEC K88 challenges [44,45]. According to what has been published on various probiotics, they show the ability to reduce epithelial permeability by increasing tight junction stability and upregulating the expression of tight junction proteins [40]. Furthermore, short-chain fatty acids (SCFAs) from probiotics, a type of postbiotic, affect trans-permeability in Caco-2 cells by similar processes, boosting their TEER values and the expression of tight junction protein genes [23,46,47].

Immunomodulatory attributes are the key in the strategy for action using postbiotics. According to numerous studies, postbiotics have immunomodulatory effects comparable to probiotics [48]. IL-10, an effective immunoregulatory cytokine with anti-inflammatory characteristics that is generated by active macrophages and T cells, suppresses T-cell proliferation and regulates pro-inflammatory cytokines, including IL-1, IL-6, and IL-8. Postbiotics can support both the innate and adaptive immune systems, leading to immunomodulatory properties, by regulating these cytokines. The present study showed that PM PD18 downregulated IL-6 and IL-8 and upregulated IL-10 expression compared with *P. gingivalis* and LPS alone. PM PD18 also showed the ability to reduce IL-6 and IL-8 and increase IL-10 secretion. Our findings are related to those of Behzadi et al., who revealed that whole-cell postbiotic *Lactobacillus* products reduce inflammation (downregulation of IL-6 and TNF-α and upregulation of IL-10) and remove free radicals in in vitro and in vivo animal models [49]. Another study found that a cell-free supernatant from *Lactobacillus reuteri* DSM 17938 increased the production of IL-10, a postbiotic cytokine with anti-inflammatory characteristics [50].

However, future studies involving animal models and clinical trials on humans are needed to determine the viability of postbiotics that support GI health.

## 4. Materials and Methods

### 4.1. Bacterial Strain and Culture Conditions

The bacterial strain *P. gingivalis* ATCC 33277 was selected as a periodontal pathogen strain that forms oral biofilms. It was obtained from the Innovation Center for Holistic Health, Nutraceuticals, and Cosmeceuticals, Faculty of Pharmacy, Chiang Mai University, Thailand. *P. gingivalis* was grown in TSB and TSA supplemented with yeast extract (Himedia, Mumbai, India), L-cysteine hydrochloride (Himedia, Mumbai, India), hemin (Sigma Aldrich, St. Louis, MO, USA), and vitamin K1 (United States Biological, Swampscott, MA, USA). It was incubated in an anaerobic chamber (Bactron 300, Sheldon MFG. Inc., Cornelius, OR, USA) with an atmosphere containing 5% H_2_, 5% CO_2_, and 90% N_2_ for 48 h.

### 4.2. Preparation of Postbiotic Metabolite of L. plantarum PD18 (PM PD18)

PM PD18 was prepared according to Butrungrod et al. [1]. Briefly, *L. plantarum* PD18 was incubated at 37 °C for 72 h after being activated in de Man Rogosa Sharpe (MRS) broth. After the supernatant was centrifuged, the pellet was discarded. Then, the cell-free supernatant (PM PD18) was sterilized by filtering it through a sterilized filter with a 0.22 µm pore diameter. The PM PD18 was neutralized to pH 7.0 ± 0.2 using 1 N NaOH and was stored at −20 °C until use.

### 4.3. Identification of PM PD18

#### 4.3.1. Gas Chromatography–Mass Spectrometry (GC–MS) Analysis

The PM compounds were analyzed using GC-MS (GC 7890-MS 5975C, Agilent technologies, Santa Clara, CA, USA) and separated on a 30 m × 0.25 mm × 0.25 µm film thickness HP-5MS capillary column. The injector port temperature was set at 250 °C. The column temperature gradually rose from 50 to 300 °C at a rate of 4 °C per minute. Helium was supplied as the carrier gas, flowing at a rate of 1 mL/min. A scan was carried out, covering a range of 40–400 amu from 2.4 to 60 min. The substance classification system relied on the National Institute of Standards and Technology’s 08 GC-MS library [51].

#### 4.3.2. High-Performance Liquid Chromatography (HPLC)

PM PD18 samples cultured for 0 and 72 h were used to quantitatively analyze 2,3,5,6-tetramethylpyrazine (TMP) by comparing them with the TMP standard (Sigma-aldrich, Burlington, MA, USA) in the standard curve. HPLC was carried out using a Shimadzu SPD-20A UV–VIS detector HPLC system (Shimadzu, Tokyo, Japan) and a Shim-pack GIST C18 column (5 µm, 4.6 × 250 mm, Shimadzu, Kyoto, Japan). The mobile phase consisted of phase A (0.1% phosphoric acid (RCl Labscan, Bangkok, Thailand) in water) and phase B (0.1% phosphoric acid in acetonitrile (RCl Labscan, Bangkok, Thailand)). The flow rate was 1.5 mL/min, and the column oven temperature was maintained at 40 °C. The detection wavelength was set at 280 nm.

### 4.4. Cell Culture

Human dermal fibroblast cells from the JCRB0075 SF-TY cell line were purchased from the Japanese Collection of Research Bioresources Cell Bank (Ibaraki, Osaka, Japan). Raw 264.7 (CL-0190) murine macrophages were obtained from Elabscience (Elabscience Biotechnology Inc., Houston, TX, USA). The cells were cultured in Dulbecco’s Modified Eagle’s Medium (DMEM) and enriched with 10% (*v*/*v*) fetal bovine serum (Gibco Laboratories, Grand Island, NY, USA), MEM Non-Essential Amino Acids Solution (100×) (MEM NEAA, Gibco Laboratories, Grand Island, NY, USA), and a 1% antibiotic–antimycotic solution (Anti-Anti, Gibco Laboratories, Grand Island, NY, USA). The cells were maintained at 37 °C in a humid environment with 5% CO_2_.

### 4.5. Safety Evaluations of PM PD18

#### 4.5.1. Cell Viability Assay

The effect of PM PD18 on the viability of the RAW 264.7 cell line was assessed using an MTT assay from a related study with modifications [52]. RAW 264.7 cells were seeded in a 96-well microplate at a density of 1 × 10^5^ cells/mL and incubated at 37 °C for 24 h with 5% CO_2_. Then, the culture medium was taken out of every well and replaced with 100 µL of DMEM with PM PD18 (100% PD18), with PM PD18 diluted to 50% (50% PD18), with PM PD18 diluted to 25% (25% PD18), or without PM PD18 (control). The plates had been incubated for 24 h before the culture media, with or without PM PD18, and were withdrawn. Then, the wells were washed with phosphate-buffered saline (PBS), and 50 µL of a 1 mg/mL MTT [(3-(4,5-dimethylthiazol-2-yl)-2,5-diphenyl tetrazolium bromide), Biobasic, New York, NY, USA] solution was added to each well. The plate was incubated at 37 °C in a humid environment with 5% CO_2_ for 4 h in the dark. The MTT solution was removed and replaced with 100 µL of dimethyl sulfoxide. The plate was gently shaken for 15 min in the dark, and the optical density (OD) was measured at 570 nm using a microplate reader (SpectraMax M3, Molecular Devices, San Jose, CA, USA). The cell viability percentage was calculated using the formula shown below:

Cell viability (%) = (OD_treated cell_/OD_control_) × 100; (1)

#### 4.5.2. Scratch Wound Healing Assay

SF-TY cells were seeded in an ibidi Culture-Insert system (ibidi GmbH, Gräfelfing, Germany) in a µ-dish at a density of 3 × 10^5^ cells/mL to produce a specific cell-free gap. Each well of the culture insert was filled with 70 µL of the cell suspension and incubated at 37 °C with 5% CO_2_. After cell confluence, the insert was taken out and washed with PBS to remove cell debris and non-attached cells. To assess its impact on cell migratory behavior, PM PD18 was diluted to a ratio of 1:2 with serum-free DMEM, added to the µ-dish, and incubated at 37 °C with 5% CO_2_. SF-TY cells, in the absence of PM PD18, served as a negative control. An inverted microscope with a camera was used to capture cell migration after 0, 24, 48, and 72 h. The photos were then subjected to digital image analysis using the ImageJ program (National Institutes of Health in Bethesda, MD, USA). The formula below was used to determine the percentage of wound closure [53]:

Percentage wound closure = [(Wound area t_0_ − Wound area t)/Wound area t_0_] × 100 (2)

### 4.6. Antiadhesion Assay

The anti-adhesive activity of PM PD18 against periodontal pathogen strains was assessed using a modified method from related research by Esteban-Fernandez et al. (2018) [38]. *P. gingivalis* was cultured, and the bacterial suspension was centrifuged at 10,000× *g* for 5 min and resuspended in non-supplemented DMEM. SF-TY cells were seeded in 24-well microplates at a density of 1 × 10^5^ cells/mL and incubated at 37 °C for 24 h with 5% CO_2_. To eliminate any FBS or antibiotic residue, the monolayer cells were gently rinsed with PBS after growing at 80% confluence. Then, bacterial suspensions with final concentrations of 10^7^ CFU/mL without PM PD18 (control) or mixed with 25 or 50% PM PD18 were added to the monolayer SF-TY cells. The plate was further incubated for 2 h. The unbound bacterial cells and medium in each well were discarded and gently rinsed with PBS. A 0.25% trypsin–EDTA solution was added to each well to remove cells and attached bacteria. Bound bacteria were cultured on each appropriate medium and incubated based on the growth conditions, as described above. The percentage of adherence inhibition (%) was calculated as shown below.

Adherence inhibition (%) = 100 × (1 − T1/T2);(3)

T1 = the number of bacteria adhered in the presence of PM PD18.

T2 = the number of bacteria adhered in the absence of PM PD18.

### 4.7. SF-TY Cells Treated with PM PD18 in Transwell Culture Insert Plate

SF-TY cells were seeded at a density of 2 × 10^5^ cells/mL on 24-well Millicell^®^ hanging cell culture inserts with a polyethylene terephthalate (PET) membrane with a pore size of 0.4 µm (EMD Millipore, Billerica, MA, USA). The cell culture medium was changed every two days. The cells and their supernatants were collected for the next study. The cells were co-cultured with LPS (1 ng/mL), *P. gingivalis,* and PM PD18 for 24 h. Then, the cell culture supernatants were collected and kept at −20 °C until use.

### 4.8. Transepithelial Electrical Resistance (TEER) Measurements

Transepithelial electrical resistance was used to measure cell monolayer integrity after treatment with LPS, *P. gingivalis,* and PM PD18. TEER was calculated by subtracting the TEER measurement from a blank transwell insert and multiplying the cell culture surface area as shown in the equation below [54]. Then, TEER was reported as a percentage compared to the value at 0 h [40].

TEER(Ω.cm^2^) = (Ω Cell Layer − Ω Blank) × Insert Surface Area cm^2^ (4)

### 4.9. Determination of Alkaline Phosphatase (ALP) Activity

The ALP activity of SF-TY cells was assessed using a previously described method with modifications [55]. Following two PBS washes, the cells cultured on the transwell insert were treated with 1 mg/mL p-nitrophenyl phosphate (p-NPP) in a 0.2 M Tris buffer and incubated at 37 °C for 30 min. To stop this process, 200 µL of 3 M NaOH was added to each well. The amount of released p-nitrophenol (p-NP) in the supernatant was measured at 405 nm using a microplate reader.

### 4.10. Effect of PM PD18 on Inflammatory Cytokines in SF-TY Cells

The cell culture supernatant from Section 4.7 was collected to study the effect of PM PD18 on the modulation of inflammatory cytokines and cell permeability after treatment with LPS. In the present study, the pro-inflammatory cytokines interleukin-6 (IL-6) and interleukin-8 (IL-8) and the anti-inflammatory cytokine interleukin-10 (IL-10) were determined using commercial enzyme-linked immunosorbent assay (ELISA) kits according to the manufacturer’s recommendations. Untreated cells served as a negative control.

### 4.11. Quantitative Real-Time Polymerase Chain Reaction (qRT-PCR) Analysis

RNA was extracted from cells and reverse-transcribed into cDNA. The obtained cDNA was used to conduct qPCR using SybrGreen to ultimately evaluate the expression rates of genes. The qPCR Master Mix SybrGreen was used for this experiment according to the manufacturer’s procedure. Quantification of inflammatory cytokines (IL-6, IL-8, and IL-10), the tight junction protein zonula occludens-1 (ZO-1), and a housekeeping gene (beta actin; β-actin) was carried out using gene-specific primers (Table 3) and analyzed using a Quant Studio 6 Flex Real-Time PCR System (Applied Biosystems, Foster City, CA, USA). Normalization of the expression data was performed using the β-actin level as a reference, and the 2^(−∆∆CT)^ method was used to determine relative mRNA levels, allowing comparisons between groups [52].

### 4.12. Western Blot (WB) Analysis

SF-TY cells were lysed in lysis buffer, and the protein concentrations in the lysate were measured using a Bradford protein assay [60]. A Sodium Dodecyl Sulfate polyacrylamide gel electrophoresis (SDS-PAGE) sample buffer was mixed with 100 mM DTT and equal amounts of protein in each sample. Then, the mixtures were heated at 95 °C for 5 min before separation using 10% SDS-PAGE (Bio-Rad, Munich, Germany) on a Mini-PROTEAN Tetra cell (Bio-Rad, Hercules, CA, USA) and electro-transferred to membranes. Membranes made of nitrocellulose (Bio-Rad, Munich, Germany) were blotted with 0.1% Tween-20 (TBST; Sigma-Aldrich, Munich, Germany) and 5% skim milk containing Tris-buffered saline for one hour at room temperature. Using primary antibodies, the membranes were incubated overnight at 4 °C, then washed with TBST and further incubated with an anti-mouse horseradish peroxidase-conjugated secondary antibody for one hour at room temperature. Blots were developed using a chemiluminescence technique. A software program in the Gel Documentation 2000 System (Bio-Rad, Hercules, CA, USA) for gel analysis was used to evaluate the blot images (Bio-Rad, Hercules, CA, USA).

### 4.13. Statistical Analysis

All data from triplicate experiments were expressed as means and standard deviations of means. To calculate statistical significance, Windows SPSS, Version 17.0 (SPSS Inc., Chicago, IL, USA), was used to perform a one-way ANOVA, followed by Tukey’s and Duncan’s post hoc tests. Statistical significance was determined at *p* < 0.05.

## 5. Conclusions

In this study, we found that the major substance in PM PD18 is TMP. PM PD18 exhibited no cytotoxic effects on RAW 264.7 cells, improved wound healing, and inhibited the adherence of *P. gingivalis* to SF-TY cells. It could preserve cell barrier integrity and could enhance the gene and protein expression of the tight junction protein ZO-1 in SF-TY cells. Furthermore, PM PD18 also exhibited immunomodulatory activities by balancing anti-inflammatory and pro-inflammatory cytokines. In conclusion, PM PD18 can be considered as a potential agent to control periodontal pathogens and their virulence factors due to its antibacterial and antibiofilm properties (as demonstrated in earlier studies).

PM PD18 also has a safety function, preserving cell membrane integrity and regulating inflammatory reactions.

## Figures and Tables

**Table 1:** HPLC analysis of major compound of PM PD18 at 0 h and 72 h of incubation.

Name	Time of Culture (h)	Retention Time (min)	Concentration (% *w*/*w*)
2,3,5,6-Tetramethylpyrazine	0	Not detected	Not detected
	72	8.896	0.100

**Table 2:** Secretion of cytokines by SF-TY cells treated with *P. gingivalis,* LPS, and PM PD18.

Sample	Secretion of Cytokines (pg/mL)		
	IL-6	IL-8	IL-10
Control	1.17 ± 0.10 ^e^	7.63 ± 0.14 ^e^	7.91 ± 0.15 ^c^
Pg	7.63 ± 0.10 ^a^	47.83 ± 0.09 ^a^	ND
LPS	3.83 ± 0.01 ^b^	22.29 ± 0.03 ^b^	ND
25% PD18	1.12 ± 0.02 ^e^	2.74 ± 0.17 ^g^	15.04 ± 0.09 ^b^
25% PD18 + Pg	1.53 ± 0.01 ^c^	7.98 ± 0.09 ^e^	6.91 ± 0.08 ^d^
25% PD18 + LPS	1.33 ± 0.06 ^d^	3.90 ± 0.08 ^f^	6.14 ± 0.09 ^e^
50% PD18	ND	4.05 ± 0.38 ^f^	92.18 ± 0.10 ^a^
50% PD18 + Pg	0.01 ± 0.00 ^f^	11.96 ± 0.13 ^c^	7.91 ± 0.07 ^c^
50% PD18 + LPS	1.15 ± 0.04 ^e^	9.12 ± 0.10 ^d^	6.60 ± 0.10 ^d^

The means ± SDs of three independent experiments are displayed. The mean individual trials within a column containing distinct letters differed significantly (*p* < 0.05) when compared with a single column. ND—not detected.

**Table 3:** The specific primers for the quantification of inflammatory cytokines and tight junction proteins using qRT-PCR.

Primer	Sequence (5′–3′)	NCBI RefSeq	Reference
IL-6	F: TGGATTCAATGAGGAGACTGCC R: CTGGCATTTGTGGTTGGGTC	NM_000600.3	[56]
IL-8	F: TCTGTGTGAAGGTGCAGTTTTG R: ATTTCTGTGTTGGCGCAGTG	NM_00584.3	[56]
IL-10	F: AGCATGGCCCAGAAATCAAG R: CGCATCCTGAGGGTCTTCAG	NM_010548	[57]
ZO-1	F: GAGTTTGATAGTGGCGTT R: GTGGGAGGATGCTGTTGT	AJ318101.1	[58]
β-actin	F: CACCCGCGAGTACAACCTTC R: CCCATACCCACCATCACACC	NM_031144.2	[59]

## Data Availability

The data presented in this manuscript are available on request from the corresponding author.
